# Two new species of the subgenus *Benbowia* Kiriakoff, 1967 (Lepidoptera, Notodontidae, *Stauropus*) from China

**DOI:** 10.3897/BDJ.14.e195460

**Published:** 2026-05-18

**Authors:** Fan Yang, Yulong Zhang, Zhaohui Pan, Min Wang

**Affiliations:** 1 South China Agricultural University, Guangzhou, China South China Agricultural University Guangzhou China; 2 Tibet Agricultural & Animal Husbandry University, Linzhi, China Tibet Agricultural & Animal Husbandry University Linzhi China

**Keywords:** Hutiaoxia, Motuo, prominent moth, *
Stauropus
*, Wuyishan, Yintiaoling

## Abstract

**Background:**

Benbowia is a distinctive subgenus within the genus Stauropus, primarily distributed in the Indomalayan realm. To date, it comprises eight species and four subspecies.

**New information:**

Two new species of the subgenus Benbowia Kiriakoff, 1967 are described: *Benbowia uncusplana* **sp. nov.**, distributed in Yunnan, Chongqing and Fujian and *Benbowia motuoensis* **sp. nov.**, distributed in Xizang, Yunnan, Nepal, India and Vietnam. Detailed diagnostic characters, descriptions and illustrations of adults and genitalia are provided.

## Introduction

The subgenus Benbowia was initially established as a genus by Kiriakoff (1967), with *Stauropus virescens* Moore, 1879, designated as its type species. In the same study, he described a new species from Sumatra (*Benbowia dudgeoni* Kiriakoff, 1967). The following year (Kiriakoff 1968), he moved Stauropus virescens loc. form takamukuanus Matsumura, 1925, which is endemic to the island of Taiwan, to this genus and treated it as a subspecies of *B. virescens*. Early taxonomic history focused primarily on the Indomalayan fauna: Dierl (1981) described a new species from Sumatra related to *B. dudgeoni* (*Benbowia elisabethae* Dierl, 1981). Subsequently, Holloway (1983) described a new species from Borneo (*Benbowia kiriakoffi* Holloway, 1983) and placed *Stauropus obscuratus* Eecke, 1929 and *B. dudgeoni* as synonyms of *B. virescens*. A series of studies by Schintlmeister substantially expanded the known diversity of the group. Schintlmeister (1993) and Schintlmeister (1997) studied notodontid specimens from the Philippines and Vietnam, describing three new species and one new subspecies (*Benbowia orientalis orientalis* Schintlmeister, 1993; *Benbowia orientalis septentrionalis* Schintlmeister, 1993; *Benbowia callista* Schintlmeister, 1997; *Benbowia camilla* Schintlmeister, 1997). Schintlmeister and Fang (2001) subsequently studied notodontid moths from China and recognised three species of *Benbowia*, including a new subspecies (*Benbowia callista xingyun* Schintlmeister & Fang, 2001). Several important nomenclatural and taxonomic changes were then made: Schintlmeister and Pinratana (2007) placed *Benbowia* as a subgenus of *Stauropus* Germar, 1812; Schintlmeister and Lourens (2010) reviewed the Philippine Notodontidae fauna and reported two new subspecies (*Benbowia orientalis distinguenda* Schintlmeister & Lourens, 2010; *Benbowia orientalis quadriga* Schintlmeister & Lourens, 2010). Most recently, Schintlmeister (2020) clarified the relationships of Borneo populations, treated *B. obscuratus* as a subspecies of *B. virescens*, *B. dudgeoni* as a synonym of *B. obscuratus* and placed *B. kiriakoffi* as a subspecies of *B. elisabethae*.

This subgenus is characterised by green, rounded fore-wings, elongated valvae, enlarged tegumen processes and an aedeagus with a trifurcate apex. Before this study, seven species and four subspecies were recognised and one species remains in *incertae sedis* (Schintlmeister 2013). Only three species and one subspecies of this subgenus have been recorded in mainland China (Schintlmeister and Fang 2001, Schintlmeister 2008).

## Materials and methods

Photos of adults were taken using a Canon EOS 200D digital camera with a LAOWA 60 mm f/2.8 2× Ultra-Macro lens. For genitalia examination, abdomens were removed and macerated in a hot 10% sodium hydroxide (NaOH) solution; photos of genitalia were taken using a Zeiss Stereo Discovery V.12 microscope. During manuscript preparation, we employed Deepseek-V4 large language models to improve the manuscript's English language. Adult and genitalia photographs were processed using Adobe Photoshop CS5 software. Terminology of adults and genitalia mainly follows Schintlmeister (2003) and Schintlmeister (2008).

### Abbreviations of the depositories used:

SCAU: South China Agricultural University, Guangzhou, China;

TAAHU: Xizang Agricultural and Animal Husbandry University, Linzhi, China;

ASPC: Alexander Schintlmeister's Personal Collections.

## Taxon treatments

### Stauropus (Benbowia) uncusplana

Zhang & Wang sp. nov.

98E1E961-C802-56A8-9598-B24A1C0828A2

253ACC9C-37EC-498B-9EFD-B1D1D83D9478

#### Materials

- **Type status:** Holotype. **Occurrence:** recordNumber: Not 242; recordedBy: Ziqi Yuan & Chuhang Qiao; sex: male; occurrenceID: BA2E587A-A365-596C-AF87-BC933997CAF6; **Location:** country: China; stateProvince: Yunnan; locality: Hutiaoxia; verbatimElevation: 1900m; **Event:** eventDate: 19 March 2024; **Record Level:** institutionCode: SCAU; ownerInstitutionCode: SCAU
- **Type status:** Paratype. **Occurrence:** recordNumber: Not 223; sex: male; occurrenceID: C1763866-1800-56ED-9503-11DF77493FCE; **Location:** country: China; stateProvince: Yunnan; county: Fuming; locality: Qinglongxia; **Event:** eventDate: 16 July 2016; **Record Level:** institutionCode: SCAU; ownerInstitutionCode: SCAU
- **Type status:** Paratype. **Occurrence:** recordedBy: Yulong Zhang & Min Wang; sex: 4 males; occurrenceID: 88B3D724-E015-58F0-8EB1-0A580650DAFA; **Location:** country: China; stateProvince: Fujian; locality: Wuyishan; verbatimLocality: 750m; **Event:** eventDate: 26 May 2021; **Record Level:** institutionCode: SCAU; ownerInstitutionCode: SCAU
- **Type status:** Paratype. **Occurrence:** recordNumber: Not 234; recordedBy: Chuyang Huang & Min Wang; sex: male; occurrenceID: 75C5AB43-6262-5A99-AE11-26CD48455021; **Location:** country: China; stateProvince: Fujian; locality: Wuyishan; verbatimLocality: 1200m; **Event:** eventDate: 17 May 2021; **Record Level:** institutionCode: SCAU; ownerInstitutionCode: SCAU
- **Type status:** Paratype. **Occurrence:** recordNumber: Not 038; recordedBy: Yulong Zhang & Min Wang; sex: male; occurrenceID: FBFC7D65-C126-5D35-A55A-1063AF8029FF; **Location:** country: China; stateProvince: Fujian; locality: Wuyishan; verbatimLocality: 750m; **Event:** eventDate: 21 May 2021; **Record Level:** institutionCode: SCAU; ownerInstitutionCode: SCAU
- **Type status:** Paratype. **Occurrence:** recordNumber: Not 222; recordedBy: Jiajia Liu & Xiaohan Ye; sex: male; occurrenceID: 7235A1AF-E93C-5D52-AC00-79F0B7696BB4; **Location:** country: China; stateProvince: Zhejiang; county: Qujiang; locality: Mt. Yaowang; **Event:** eventDate: 11 March 2023; **Record Level:** institutionCode: SCAU; ownerInstitutionCode: SCAU
- **Type status:** Paratype. **Occurrence:** recordNumber: Not 246; recordedBy: Jiajia Liu & Xiaohan Ye; sex: male; occurrenceID: 336EA72C-F7ED-5BA9-9856-8A63288DCF07; **Location:** country: China; stateProvince: Zhejiang; county: Qujiang; locality: Mt. Yaowang; **Event:** eventDate: 25 February 2026; **Record Level:** institutionCode: SCAU; ownerInstitutionCode: SCAU

#### Description

Male (Fig. 1a, b, c, d, e). Wingspan 36-39 mm, fore-wing length 15-19 mm. Antennae are bipectinate, brown and filiform apically. Palpi are brown. The head and thorax are covered with green hair. The abdomen is dorsally brown, basal tufts are green, blackish-brown-edged; tergal tufts are blackish-brown in the 1^st^-5^th^ segments; the anal tuft is green, with light brown or yellowish-white scales. The fore-wing ground colour is green, scattered with brown scales; the inner, discal, outer, subterminal and terminal lines are composed of brown spots with pale yellowish-white. The inner line is double, slightly curved, oblique outwards and filled in with pale yellowish-white; the discal line is indistinct and serrated; the outer line is double, curved in the middle and ending near the tornus and filled in with yellowish-white; the subterminal line is indistinct, with distinct brown spots near the anterior margin; the terminal line is composed of brown cell spots. Cilia are light brown mixed with yellowish-white. The hind-wing is pale brown, the costa is broadly green with three parallel brown fasciae.

Male genitalia (Figs 3a, b, c, d, e, 5a, b, c, d, e). The 8^th^ tergite has a medially arcuate margin with serrated lateral sides. The 8^th^ sternite is bilobed posteriorly, with a sclerotised extension towards the lateral sides of uniform thickness and the sclerotised extension towards the base is slender. Socii are short and robust, with a pointed apex and a serrated lateral lobe. The tegumen is broad, with a small basal process. Valvae are long and narrow, with an enlarged apex bearing a subapical pollex. The saccular process is broad, with several large teeth. The phallus is slightly curved distally, with an enlarged bulb and two sclerotised hook-shaped processes at the apex.

#### Diagnosis

The new species is externally similar to *B. callista* (Figs 1f, 2a), but can be distinguished by the more prominent inner line on the fore-wing. The male genitalia exhibit distinct differences: socii are shorter with a pointed apex, contrasting with those of *B. callista*, which are longer and distally broader with a rounded apex (Figs 3f, 4a); the tegumen is narrower and the basal process of the tegumen is extremely slender in *B. uncusplana* sp. nov., but broader and irregularly shaped in *B. callista*.

#### Etymology

The specific name is derived from the morphological term uncus and the Latin suffix -*plana*, meaning flat, referring to the flattened uncus lobes.

#### Distribution

China: Yunnan, Chongqing, Fujian, Zhejiang.

#### Taxon discussion

In the original description of *B. callista xingyun*, there are seven paratypes from Hutiaoxia, Yunnan (GU (02-01) and GU 55-05) and Chongqing. Based on the illustration (Schintlmeister and Fang 2001: fig. 39), these paratypes are hereby assigned to this new species.

### Stauropus (Benbowia) motuoensis

Zhang & Pan sp. nov.

99A25E1D-9623-56EF-ABD5-5D6C869FEC33

651A194A-989B-4762-AC7B-8C64F5095F97

#### Materials

- **Type status:** Holotype. **Occurrence:** recordNumber: STS-82148; recordedBy: Zhaohui Pan; sex: male; occurrenceID: 6FEC35DC-5275-5063-AA39-60A589814673; **Location:** country: China; stateProvince: Xizang Auton. Reg.; county: Motuo; locality: 80K; verbatimElevation: 2115 m; **Event:** eventDate: 19 July 2017; **Record Level:** institutionCode: TAAHU; ownerInstitutionCode: TAAHU
- **Type status:** Paratype. **Occurrence:** recordNumber: STS-76258; recordedBy: Zhaohui Pan; sex: male; occurrenceID: 24E959F2-38FD-56F1-A259-343FF2105261; **Location:** country: China; stateProvince: Xizang Auton. Reg.; county: Motuo; verbatimElevation: 1111 m; **Event:** eventDate: 06 July 2018; **Record Level:** institutionCode: TAAHU; ownerInstitutionCode: TAAHU
- **Type status:** Paratype. **Occurrence:** recordNumber: STS-43762; recordedBy: Zhaohui Pan; sex: male; occurrenceID: 07AC5DAD-3A10-5EC5-9B4D-B6DEC6D4FCA0; **Location:** country: China; stateProvince: Xizang Auton. Reg.; county: Motuo; locality: 80K; verbatimElevation: 2115 m; **Event:** eventDate: 09 July 2018; **Record Level:** institutionCode: TAAHU; ownerInstitutionCode: TAAHU
- **Type status:** Paratype. **Occurrence:** recordNumber: STS-1125; recordedBy: Zhaohui Pan; sex: male; occurrenceID: D3701999-16F4-5397-B3B5-55457CA9DEB8; **Location:** country: China; stateProvince: Xizang Auton. Reg.; county: Motuo; locality: Hanmi; verbatimElevation: 2123 m; **Event:** eventDate: 19 July 2013; **Record Level:** institutionCode: SCAU; ownerInstitutionCode: TAAHU

#### Description

Male (Fig. 2b, c). Wingspan 40-42 mm, fore-wing length 19-20 mm. Antennae are bipectinate, brown and filiform apically. Palpi are brown. The head and thorax are covered with green hair; the abdomen dorsally is light brown with green basal tufts edged with blackish-brown; the tergal tufts are blackish-brown in the 1^st^-5^th^ abdomen segments; the anal tuft is green mixed with yellowish-white and brown scales. The fore-wing ground colour is green, scattered with brown scales; the inner, discal, outer, subterminal and terminal lines are composed of brown spots with yellowish-white; the inner line is double, serrated, oblique outwards, filled in with yellowish-white; the discal line is indistinct, serrated; the outer line is double, curved in the middle and ending near the tornus, filled in with yellowish-white; the subterminal line is indistinct, edged with yellowish-white with distinct brown spots near the anterior margin; the terminal line is composed of brown interveinal spots. Cilia are light brown mixed with yellowish-white. The hind-wing is pale brown, with a broadly green costa bearing three parallel brown fasciae.

Male genitalia (Figs 4b, c, 6b). The 8^th^ tergite is posteriorly concave, flanked by denticulate, semicircular processes. The 8^th^ sternite bears a bilobed process; its sclerotised extension towards the lateral sides is short and the sclerotised extension towards the base is slender. Socii are short and robust, with an ellipsoid apex, with a row of small spines ventrally. The tegumen is narrow, with a small process at the base. The valvae are thick, the apex enlarged and serrated, with a short pollex subapically. The saccular process bears a lobe-shaped process with serrated margins. The phallus is slender, with an apex that has two widely sclerotised hook-shaped processes.

#### Diagnosis

The new species is similar to *B. camilla* (Fig. 2d), but can be distinguished by the following characters: the inner line on the fore-wing is more prominent, the triangular brown patch near the apex is broader and the second brown fascia on the hind-wing costal area is more prominent. The male genitalia can be distinguished by the following characters: socii are rounder, without ventral lobes; the valvae are broader in the middle, apically rectangular with a smaller pollex (apically more rounded in *B. camilla*); the aedeagus is longer, with a trifurcate apex and its spines are basally broader than those of *B. camilla*; the 8^th^ tergite is rounded and U-shaped concave distally (V-shaped in *B. camilla*) (Figs 4d, 6c).

#### Etymology

The specific epithet *motuoensis* refers to the type locality, Motuo County, Xizang Autonomous Region.

#### Distribution

China: Xizang Autonomous Region (Motuo), Yunnan (Yunxian-Daxing); Nepal; India (W Bengal, Bhimtal); N Vietnam.

## Supplementary Material

XML Treatment for Stauropus (Benbowia) uncusplana

XML Treatment for Stauropus (Benbowia) motuoensis

## Figures and Tables

**Figure 1a.**
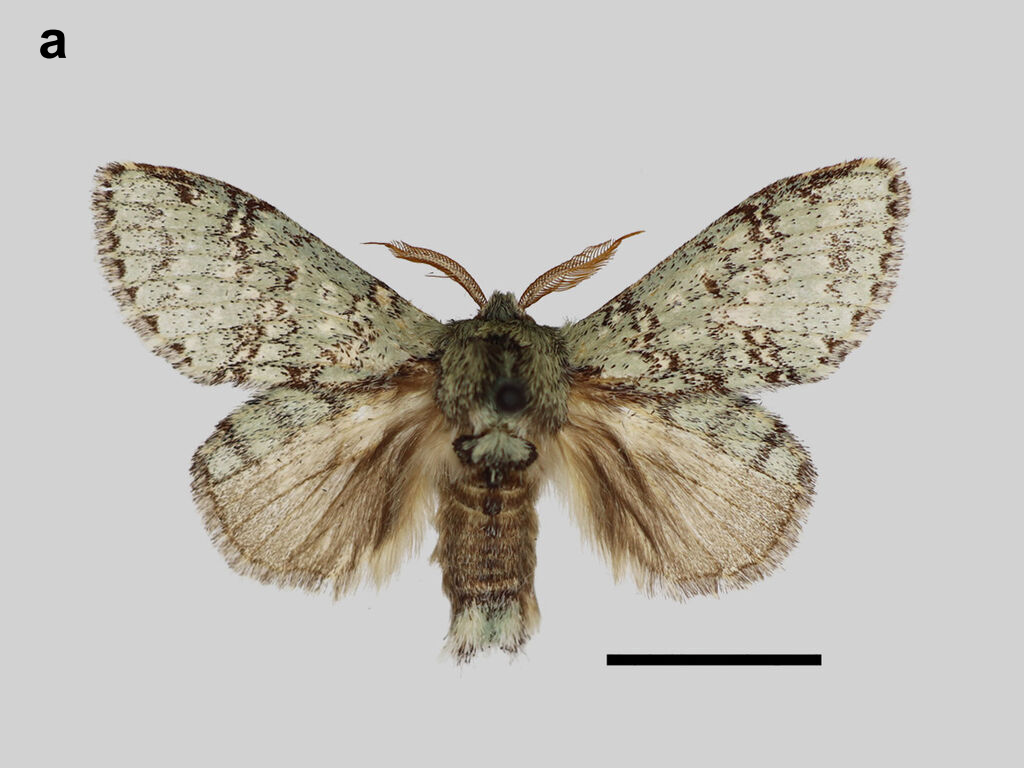
*Benbowia uncusplana* sp. nov., holotype, ♂, Yunnan, Not242;

**Figure 1b.**
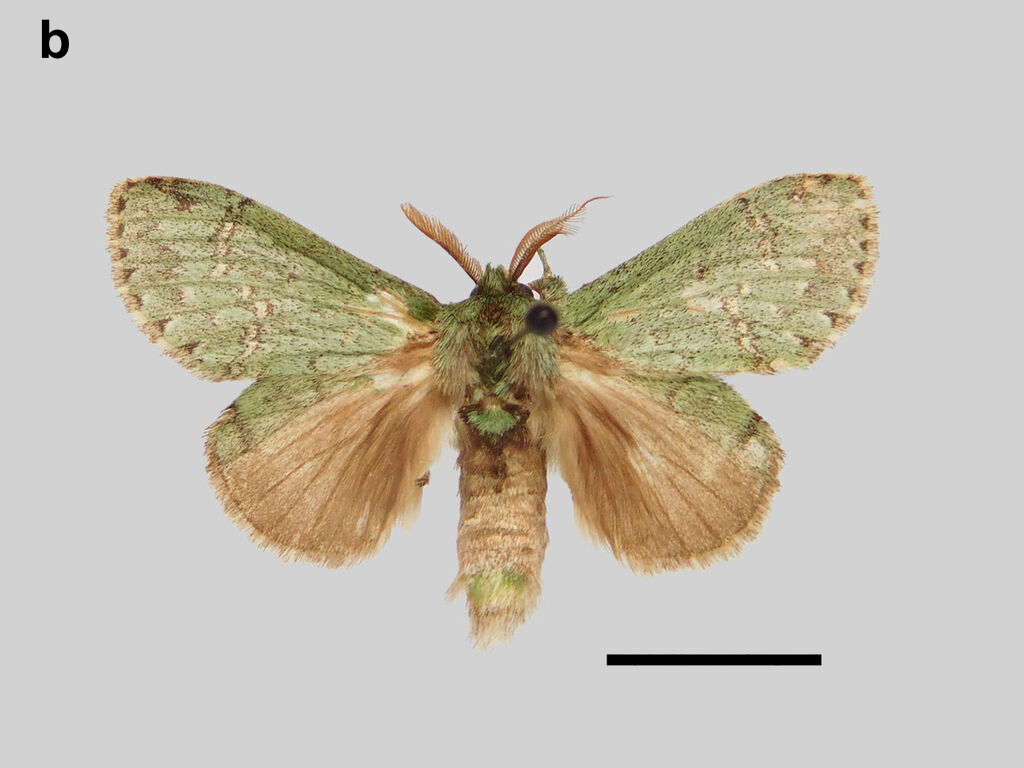
*Benbowia uncusplana* sp. nov., paratype, ♂, Yunnan, Not223;

**Figure 1c.**
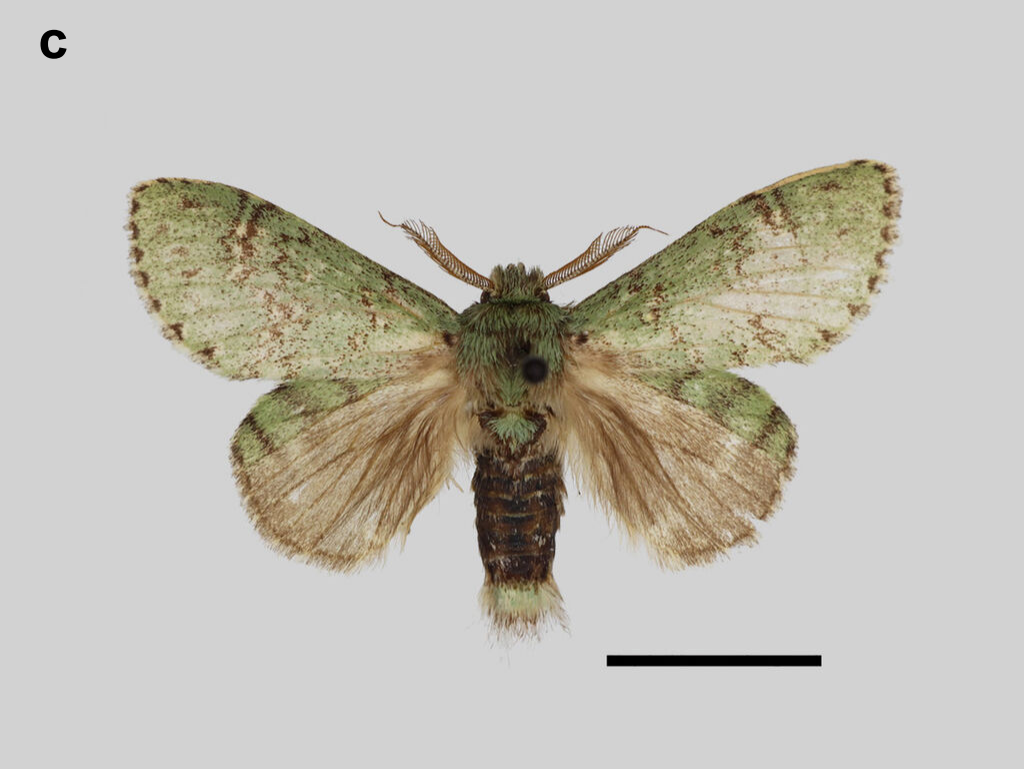
*Benbowia uncusplana* sp. nov., paratype, ♂, Fujian, Not234;

**Figure 1d.**
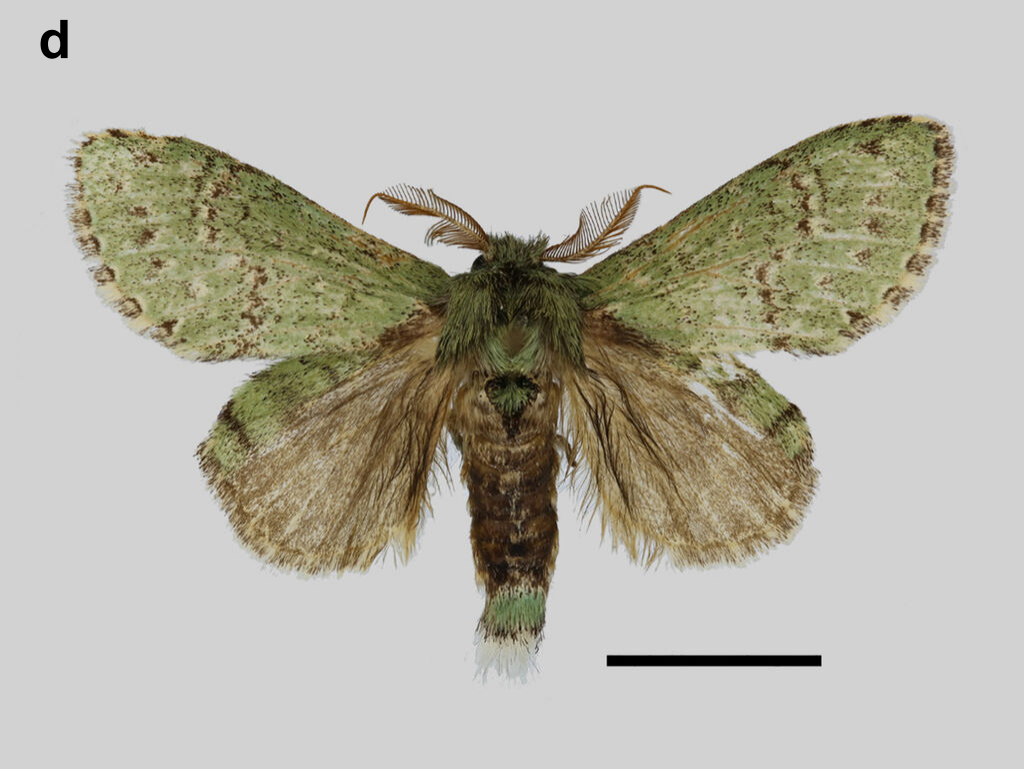
*Benbowia uncusplana* sp. nov., paratype, Chongqing, YTL02;

**Figure 1e.**
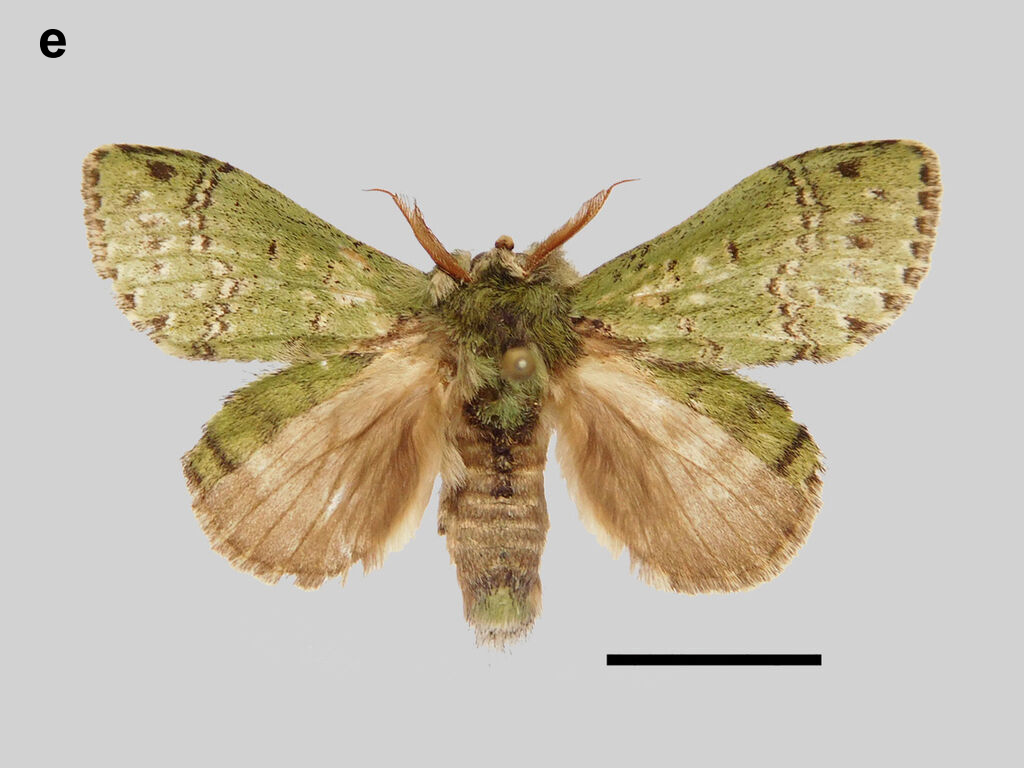
*Benbowia uncusplana* sp. nov., paratype, ♂, Zhejiang, Not222;

**Figure 1f.**
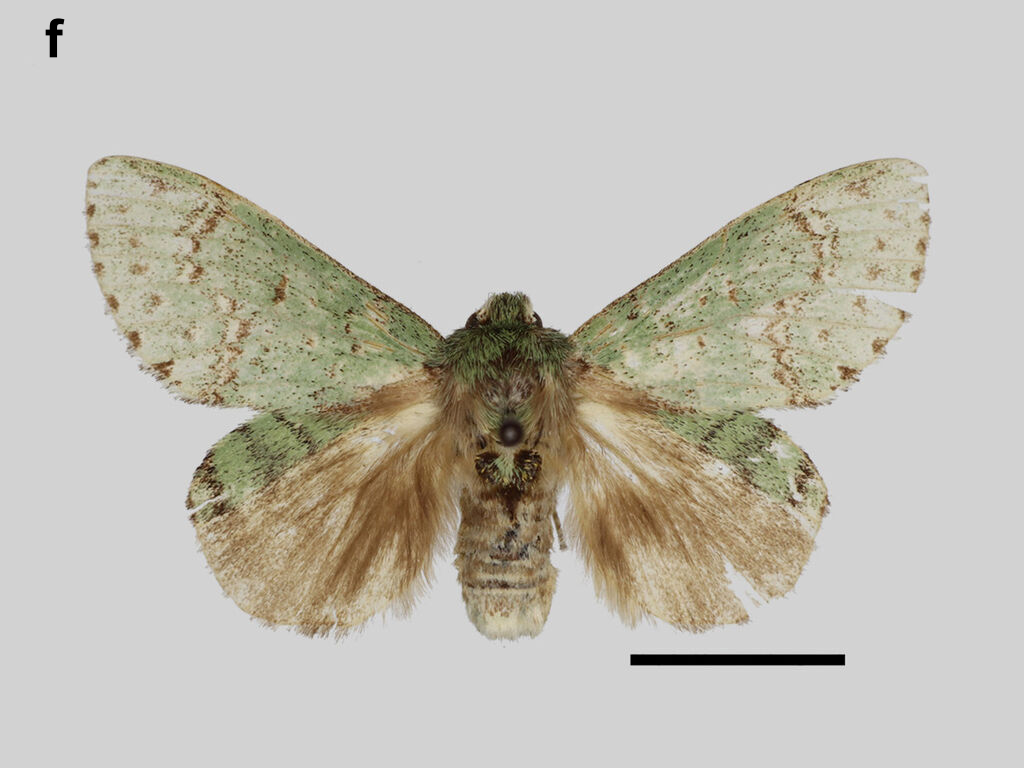
*Benbowia callista xingyun*, ♂, Sichuan, Not224.

**Figure 2a.**
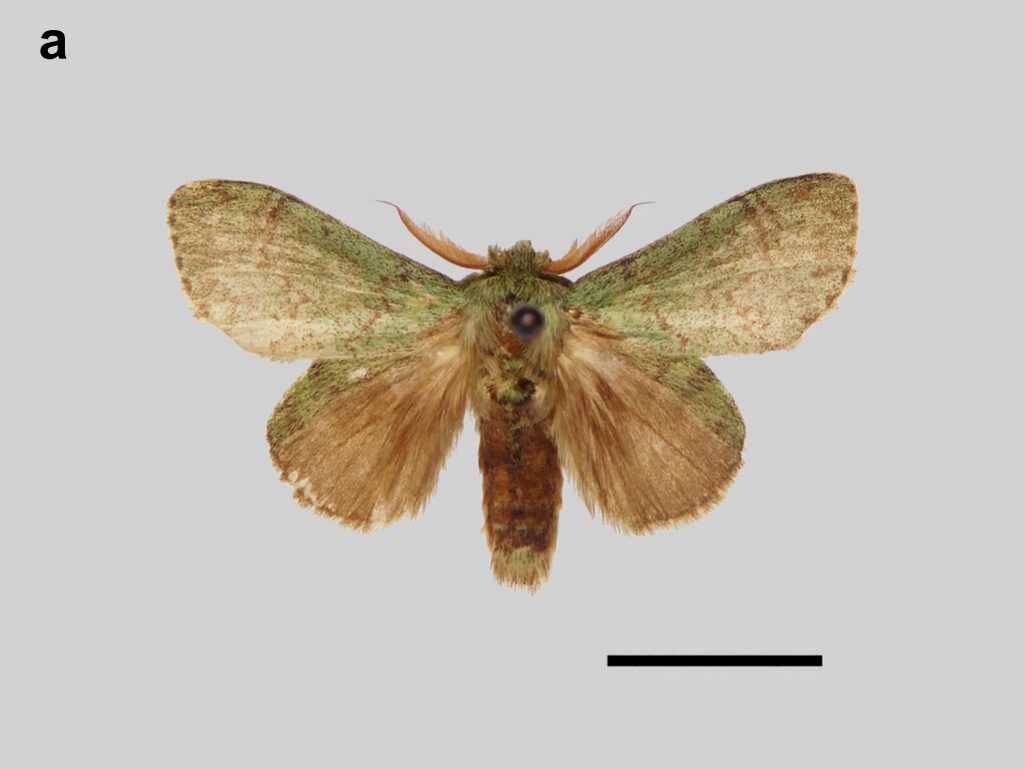
*Benbowia callista*, ♂, Zhejiang, Not225;

**Figure 2b.**
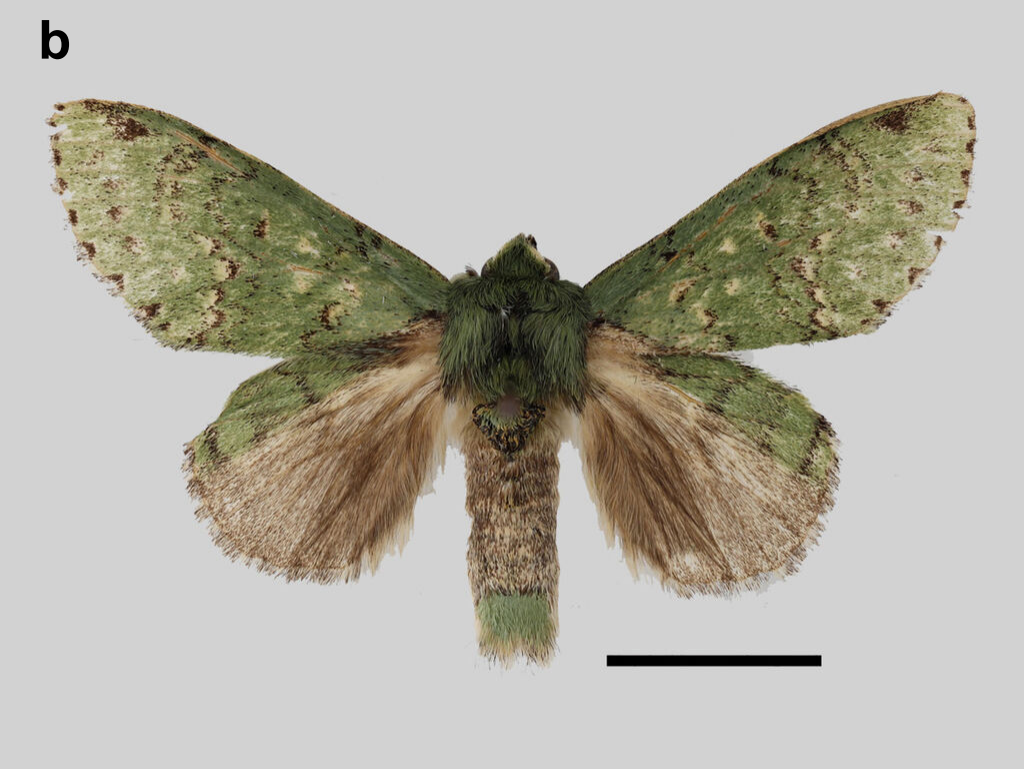
*Benbowia motuoensis* sp. nov., holotype ♂, Xizang, STS-82148;

**Figure 2c.**
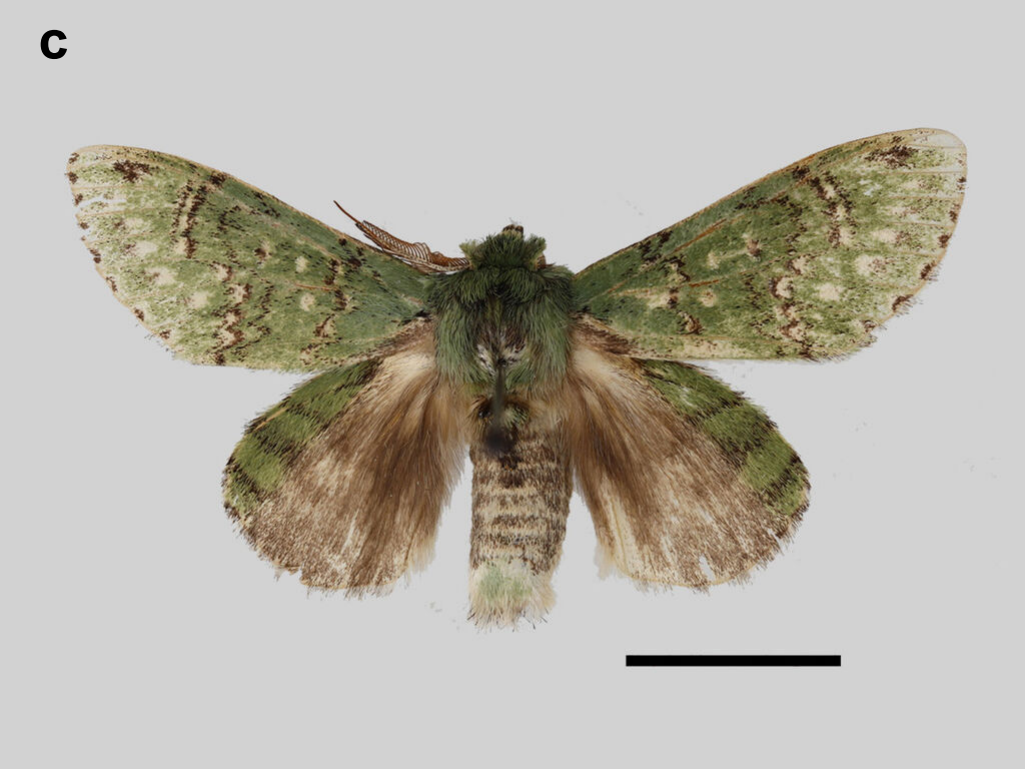
*Benbowia motuoensis* sp. nov., paratype, Xizang, STS-76258;

**Figure 2d.**
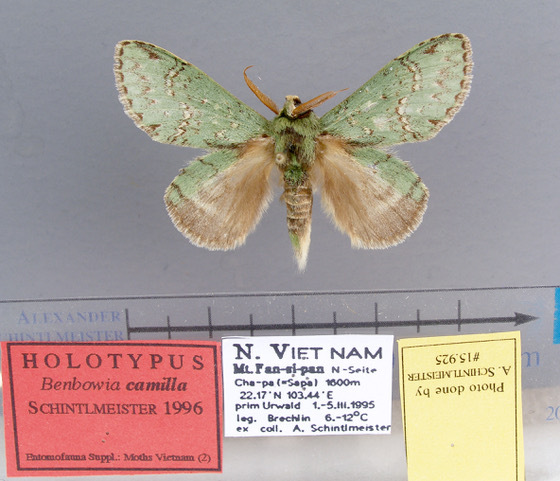
*Benbowia camilla*, ♂, holotype, Vietnam, ASPC.

**Figure 3a.**
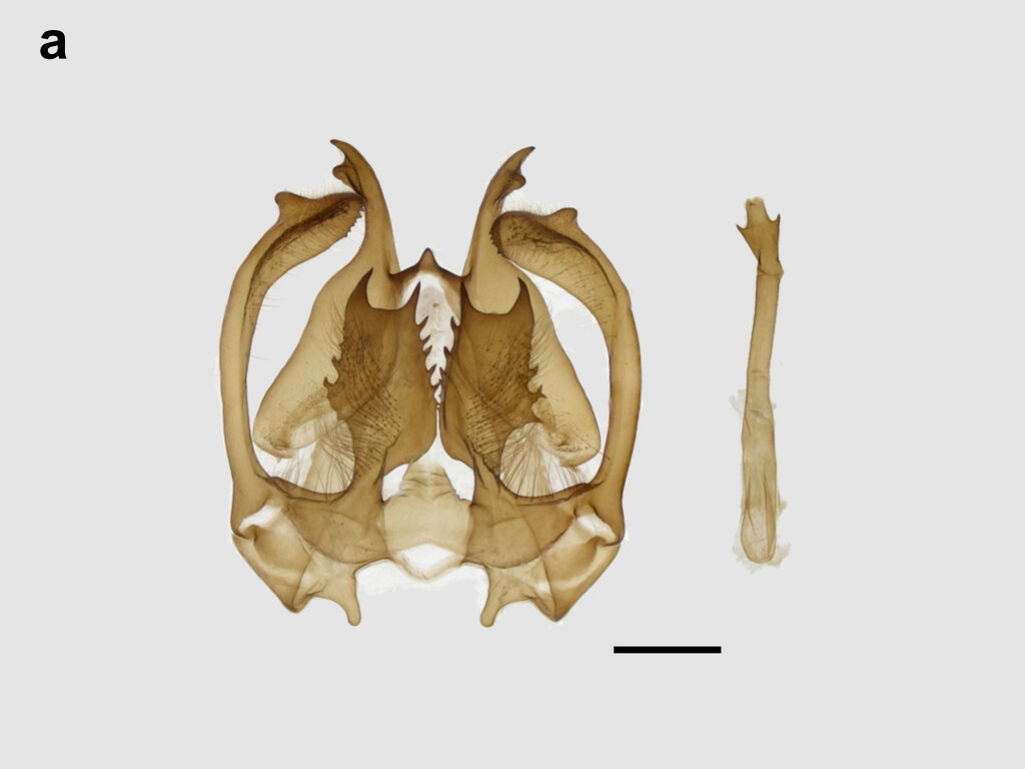
*Benbowia uncusplana* sp. nov., holotype, ♂, Yunnan, Not242;

**Figure 3b.**
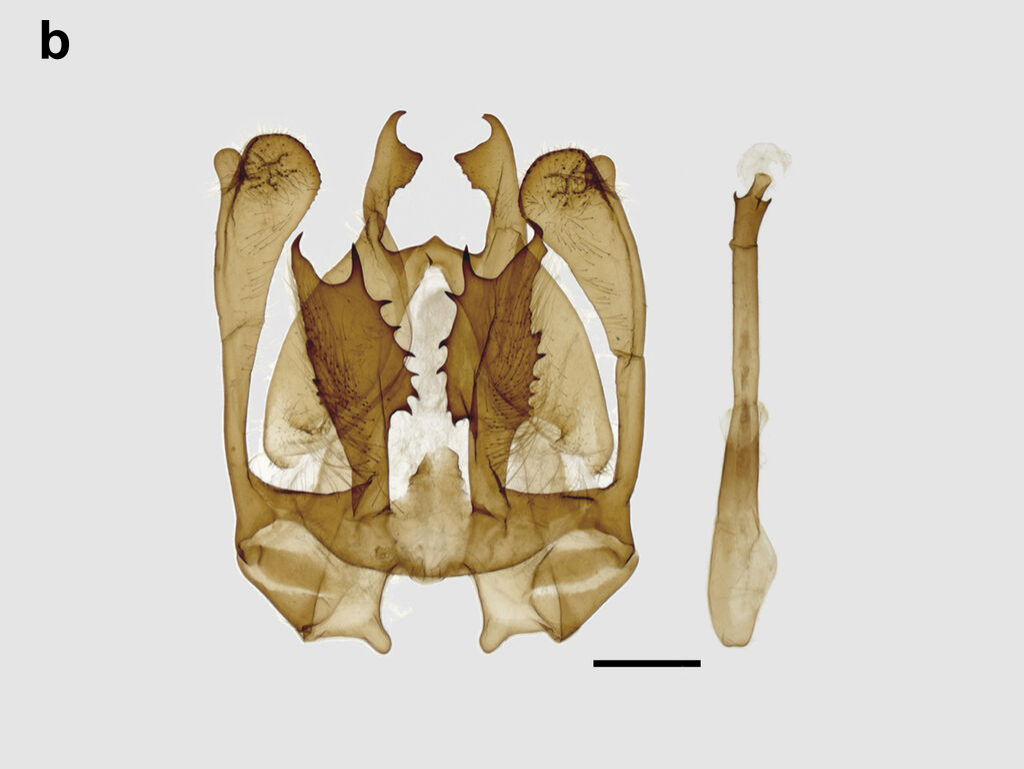
*Benbowia uncusplana* sp. nov., paratype, ♂, Yunnan, Not223;

**Figure 3c.**
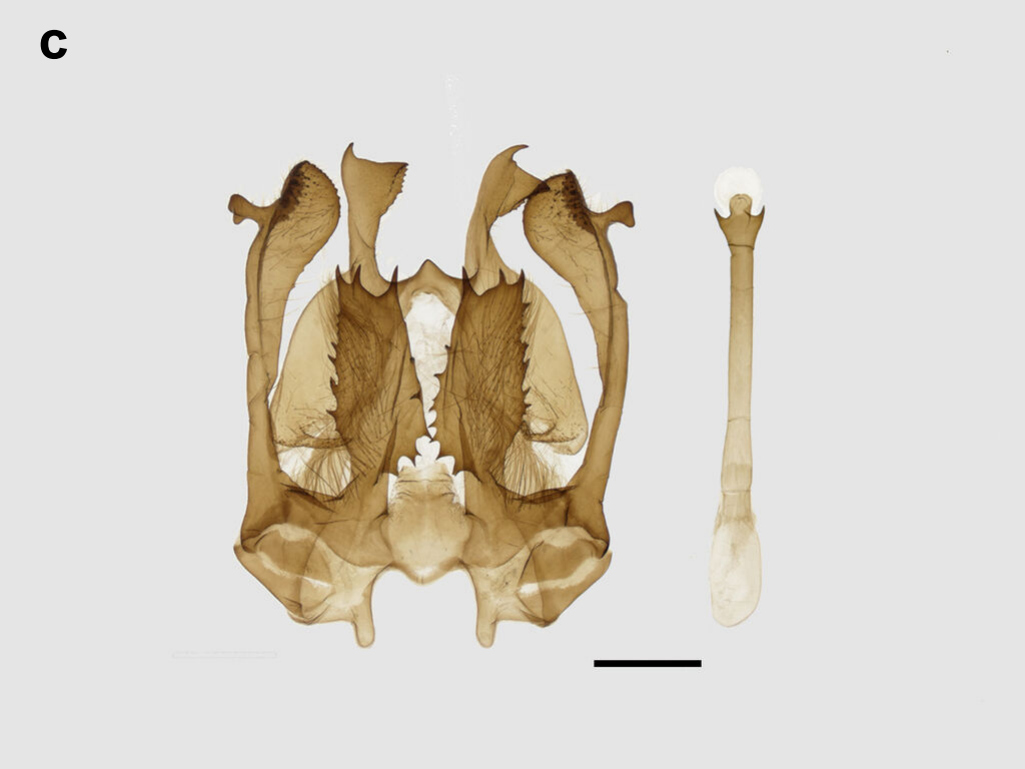
*Benbowia uncusplana* sp. nov., paratype, ♂, Fujian, Not234;

**Figure 3d.**
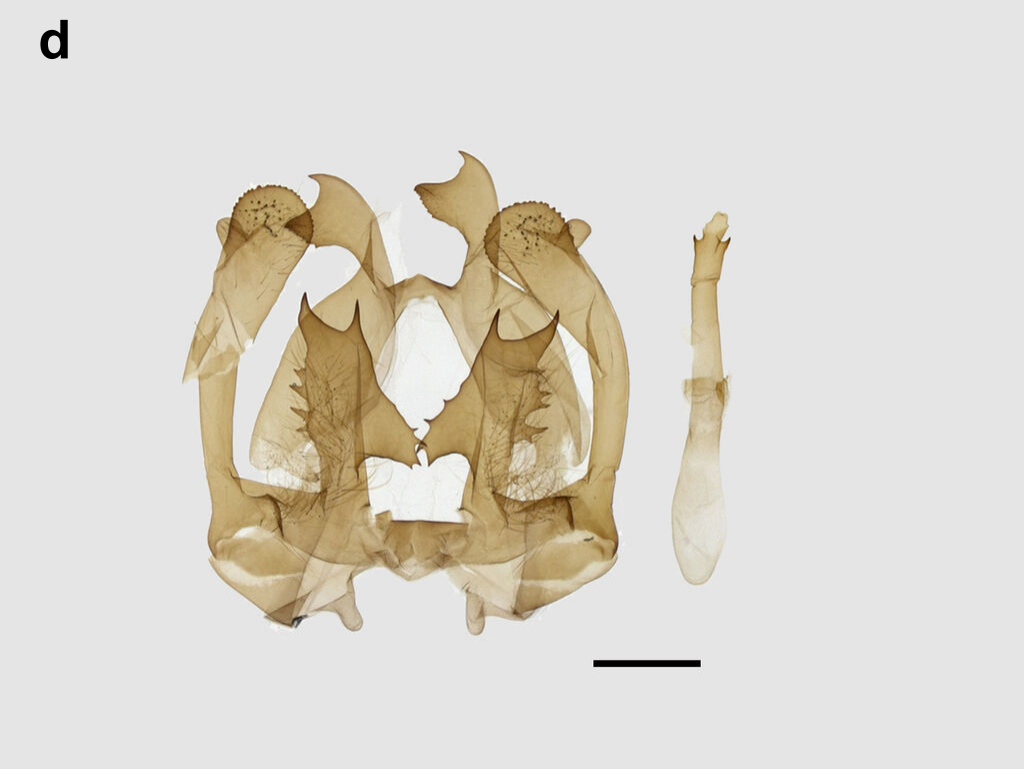
*Benbowia uncusplana* sp. nov., paratype, Chongqing, YTL01;

**Figure 3e.**
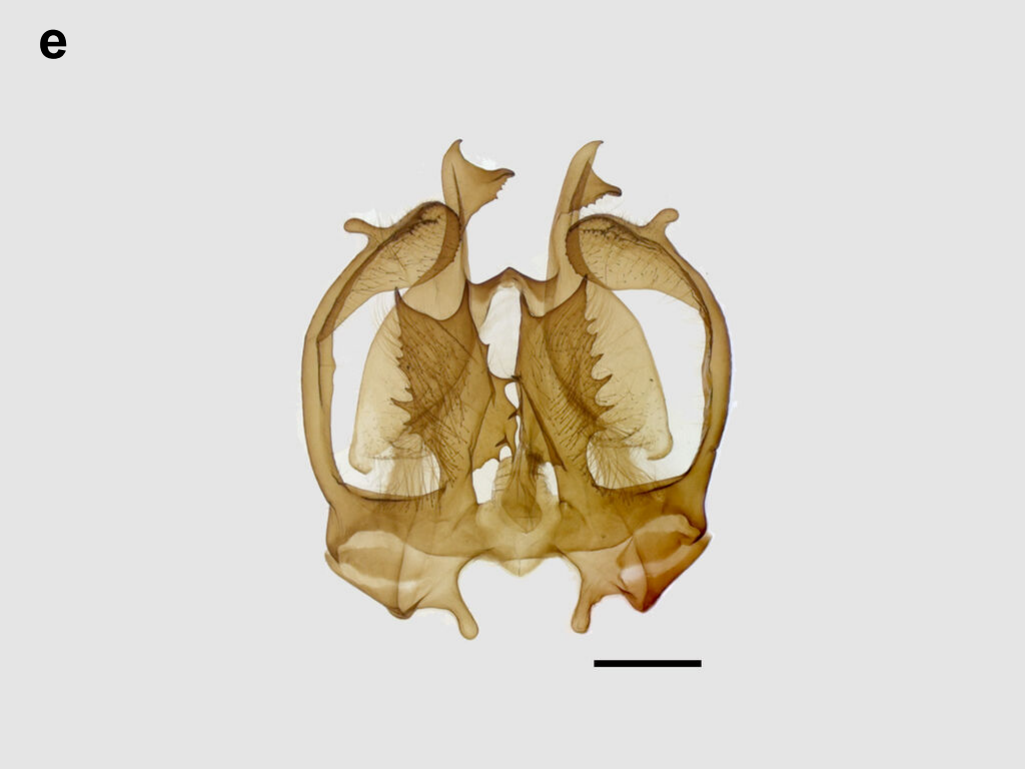
*Benbowia uncusplana* sp. nov., paratype, ♂, Zhejiang, Not222, phallus missing;

**Figure 3f.**
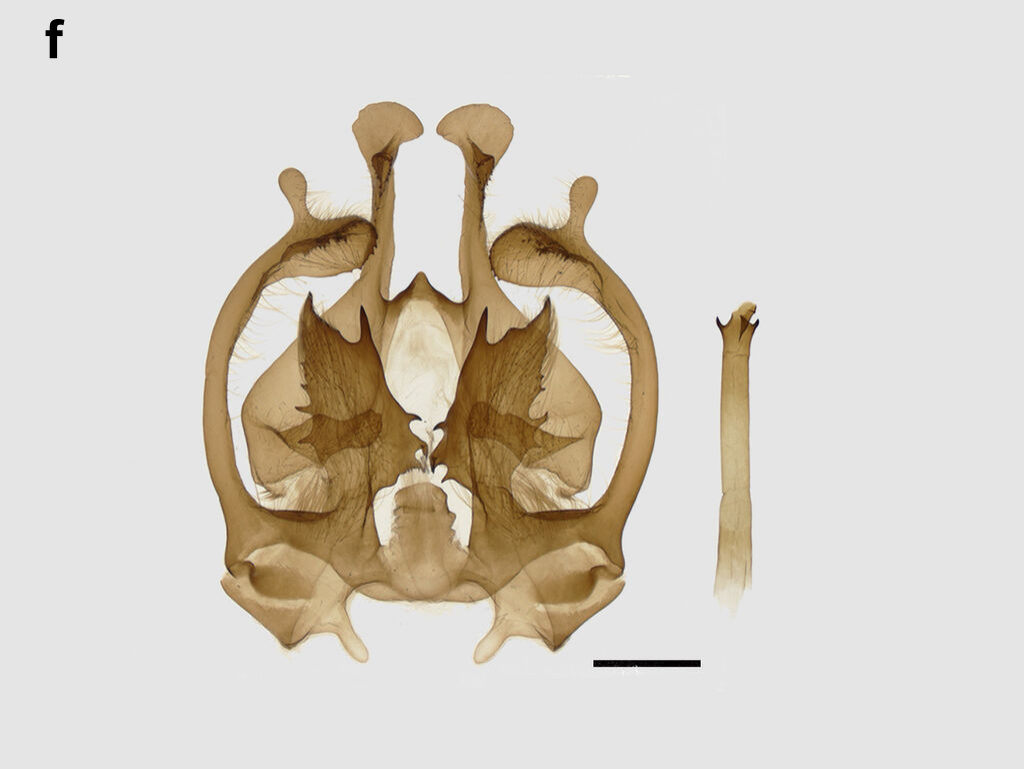
*Benbowia callista xingyun*, Sichuan, Not224.

**Figure 4a.**
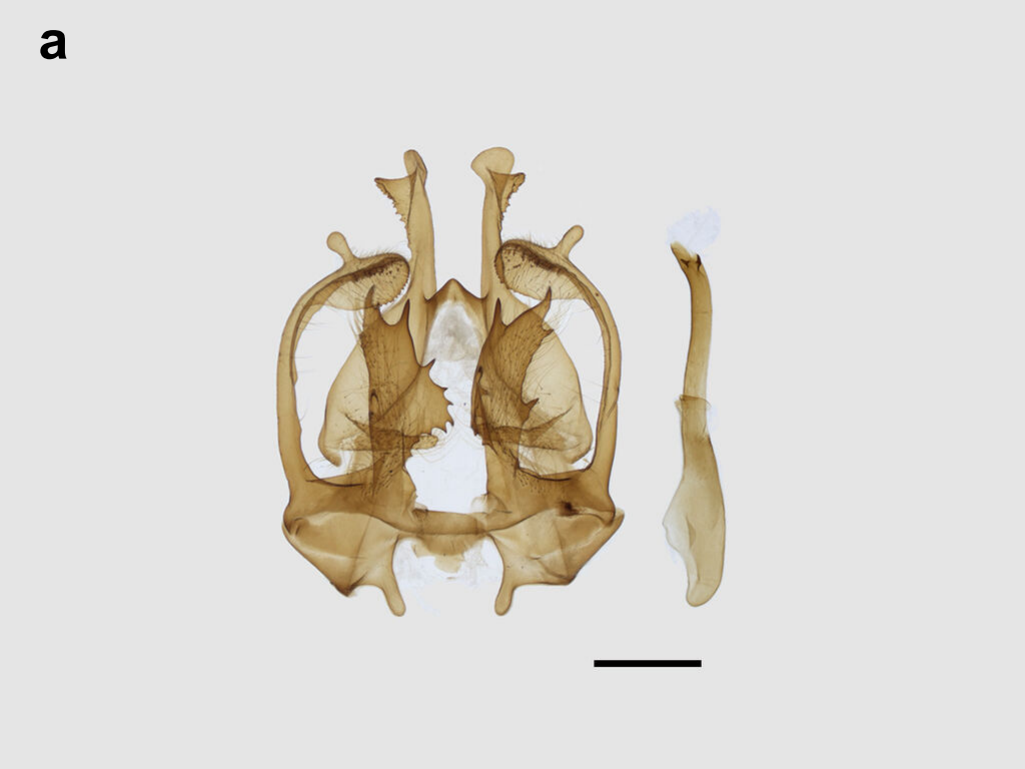
*Benbowia callista*, ♂, Zhejiang, Not225;

**Figure 4b.**
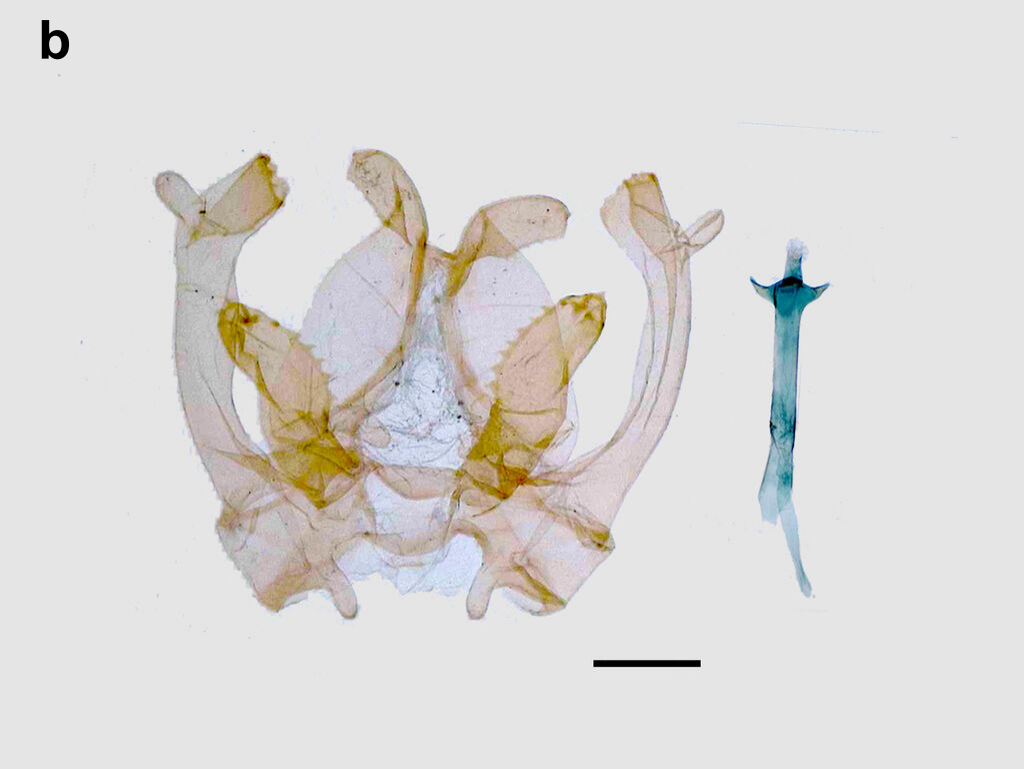
*Benbowia motuoensis* sp. nov., holotype, ♂, Xizang, STS-82148;

**Figure 4c.**
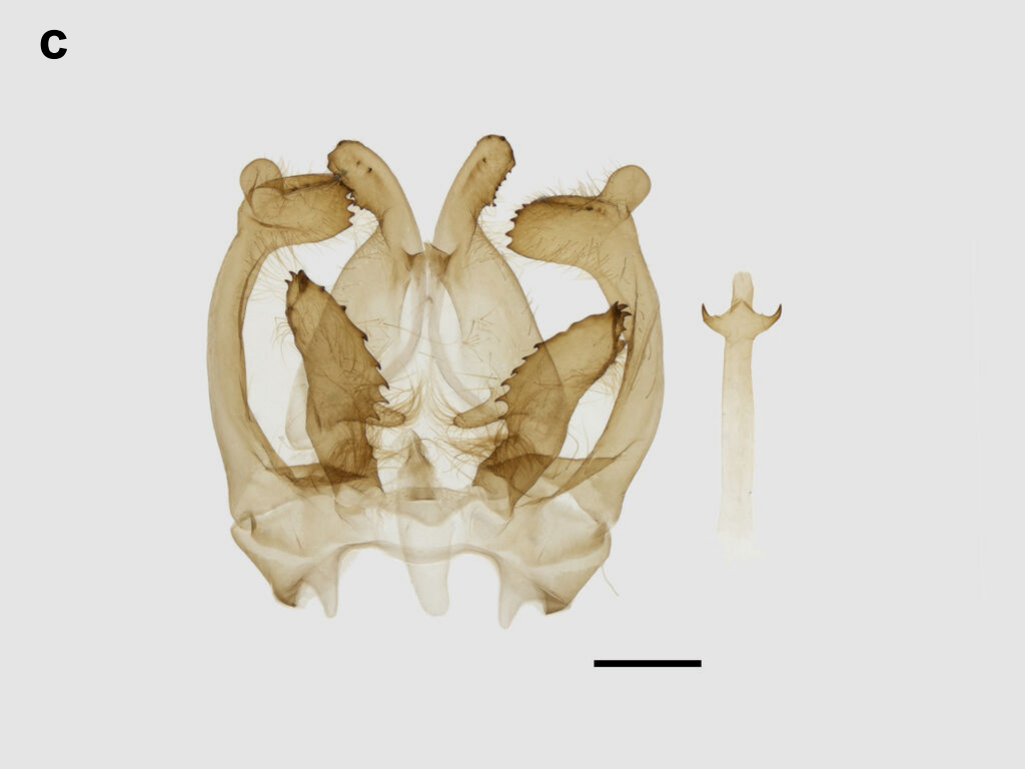
*Benbowia motuoensis* sp. nov., paratype, Xizang, STS-1125;

**Figure 4d.**
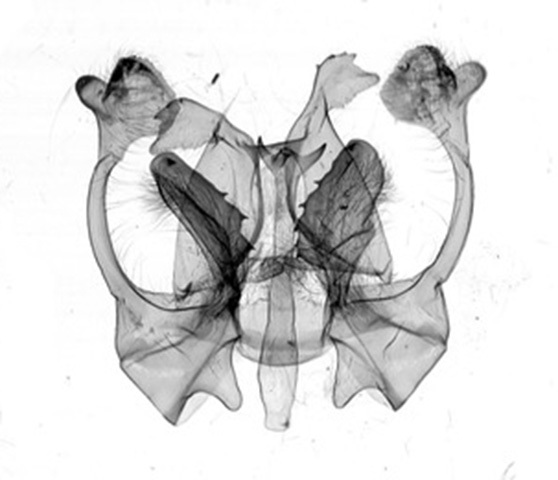
*Benbowia camilla*, ♂, paratype, Vietnam, ASPC.

**Figure 5a.**
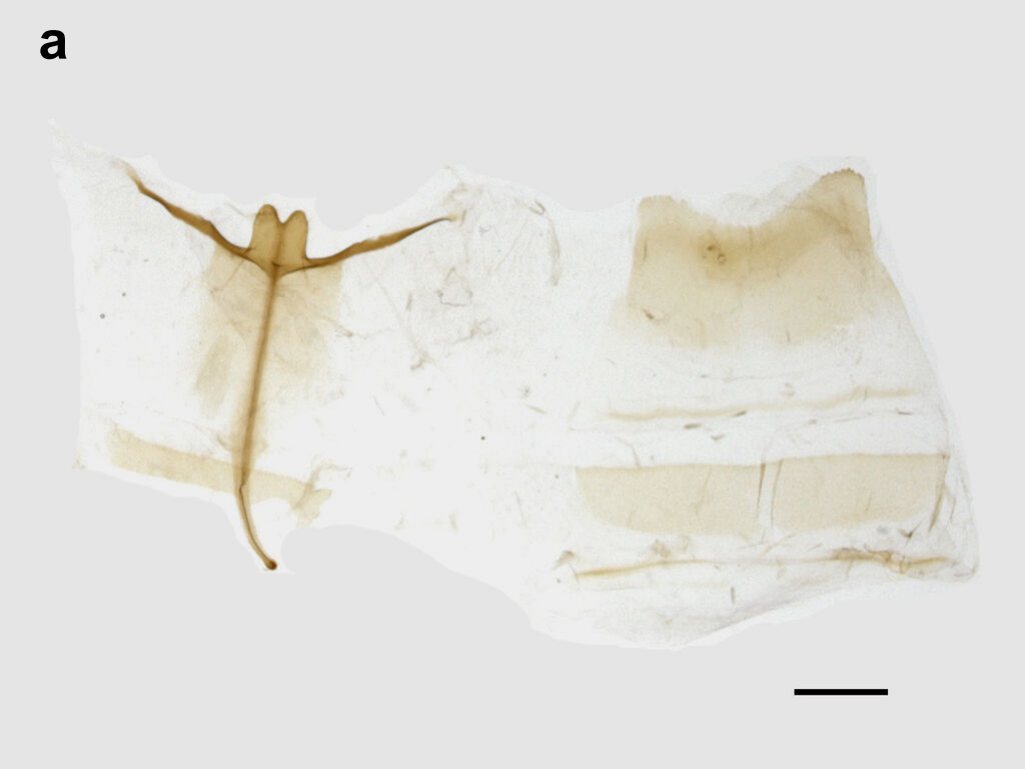
*Benbowia uncusplana* sp. nov., holotype, ♂, Yunnan, Not242;

**Figure 5b.**
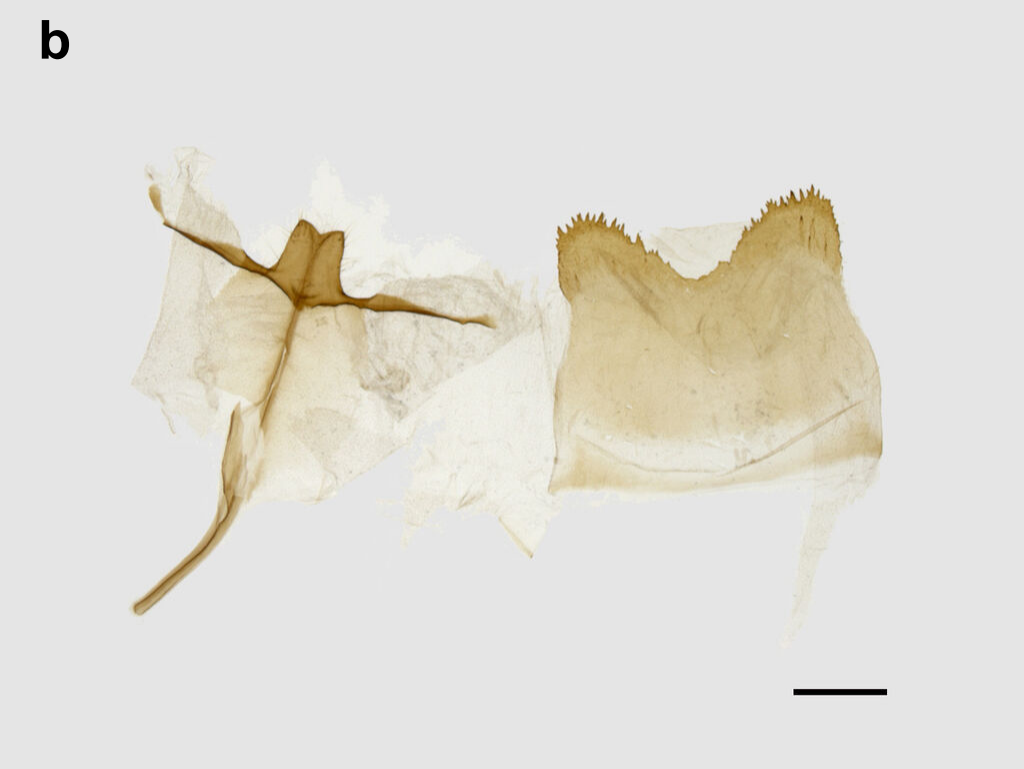
*Benbowia uncusplana*, paratype, ♂, Yunnan, Not223;

**Figure 5c.**
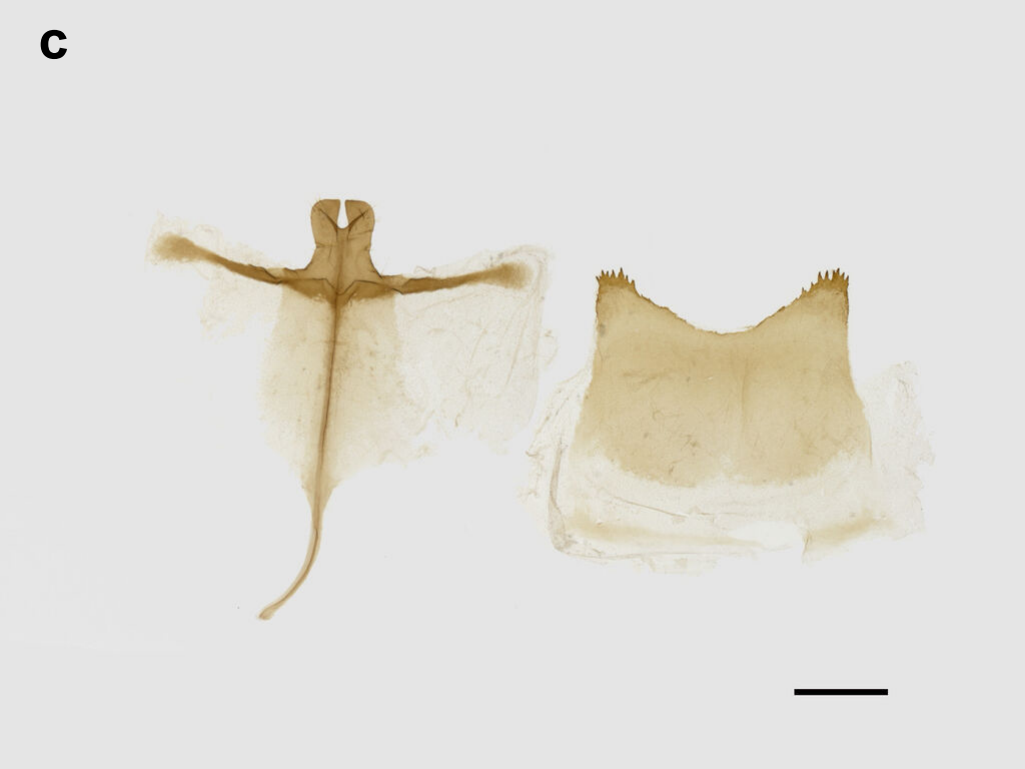
*Benbowia uncusplana*, paratype, ♂, Fujian, Not234;

**Figure 5d.**
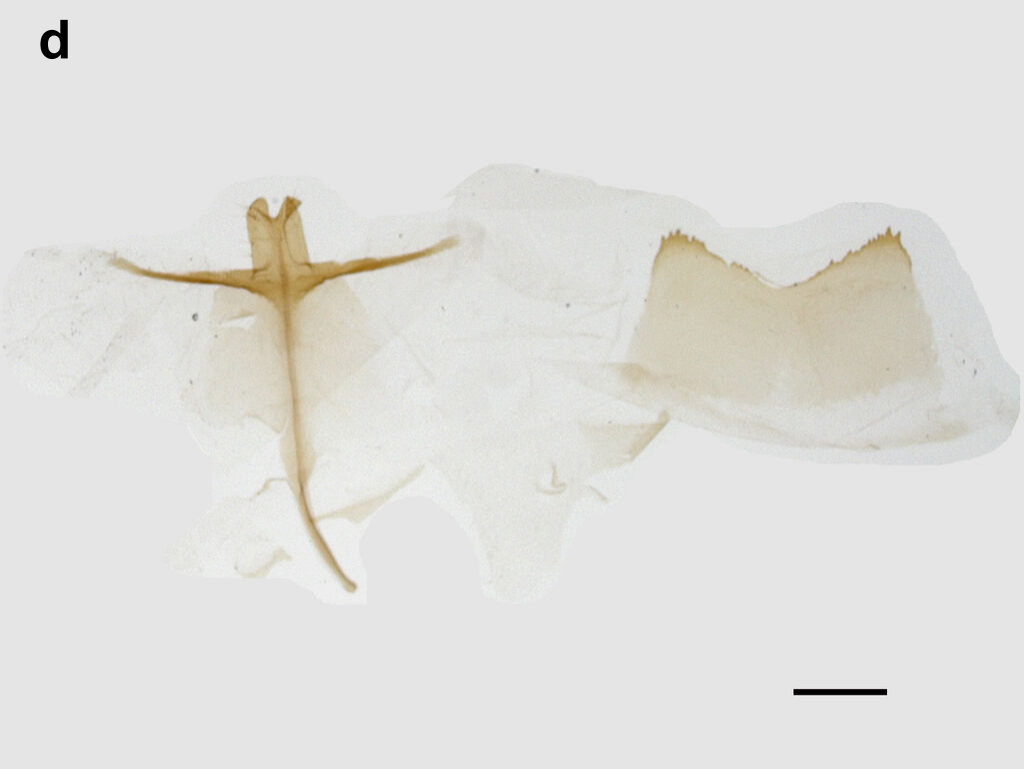
*Benbowia uncusplana*, paratype, ♂, Chongqing, YTL01;

**Figure 5e.**
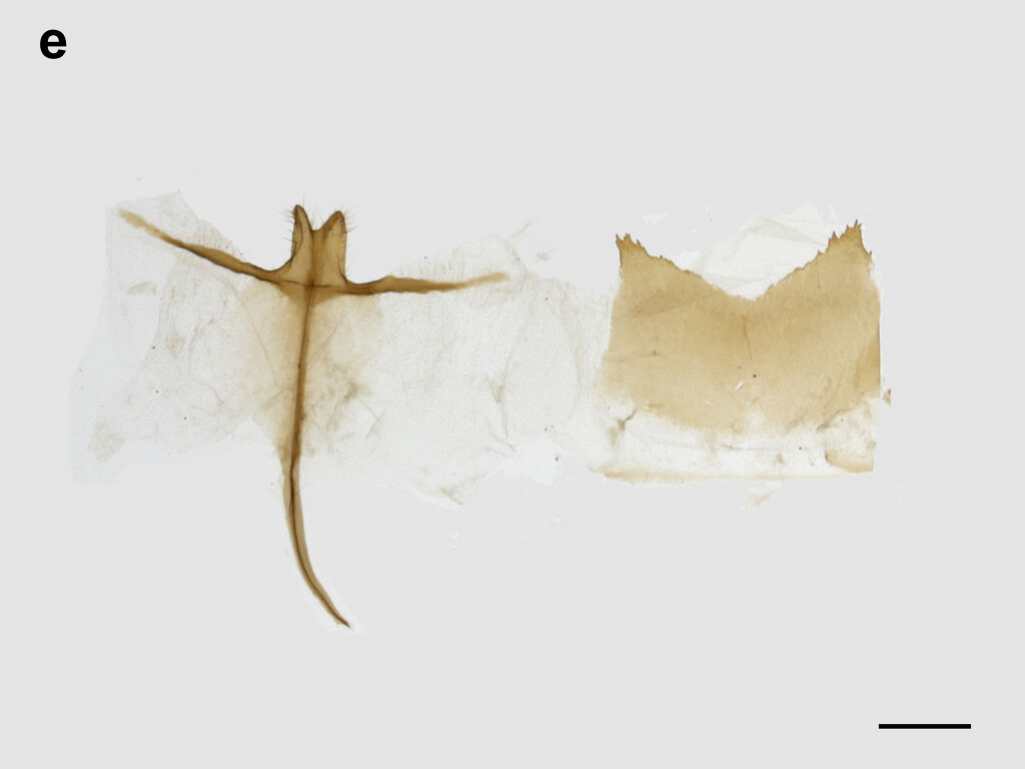
*Benbowia uncusplana*, paratype, ♂, Zhejiang, Not222;

**Figure 5f.**
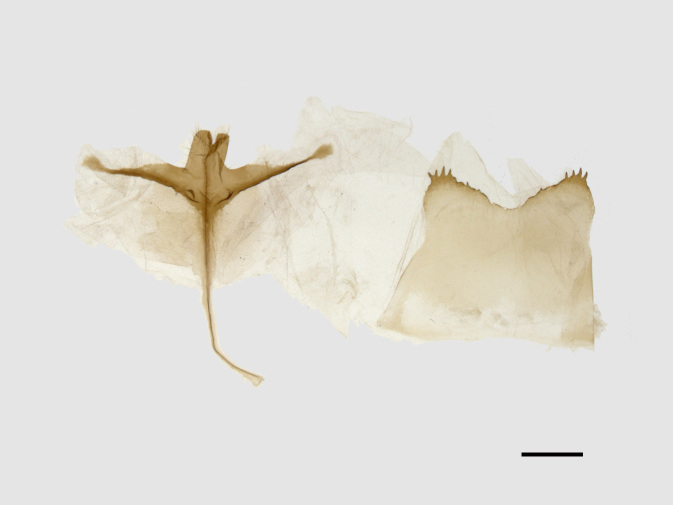
*Benbowia callista xingyun*, ♂, Sichuan, Not224.

**Figure 6a.**
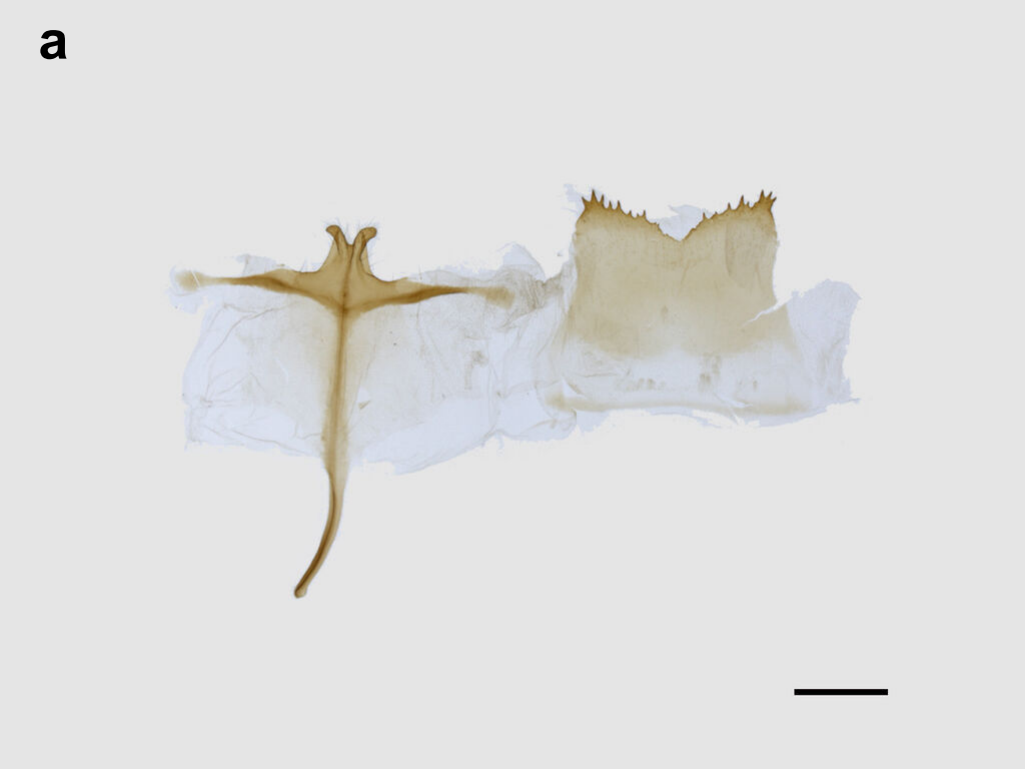
*Benbowia callista*, ♂, Zhejiang, Not225;

**Figure 6b.**
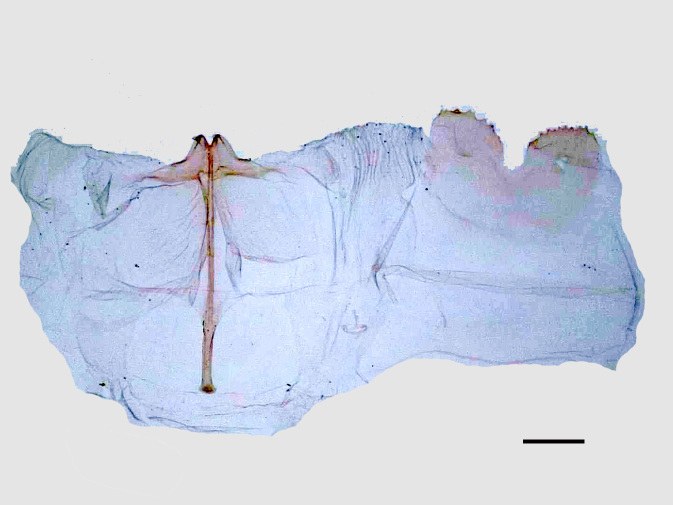
*Benbowia motuoensis* sp. nov., holotype, ♂, Xizang, STS-82148;

**Figure 6c.**
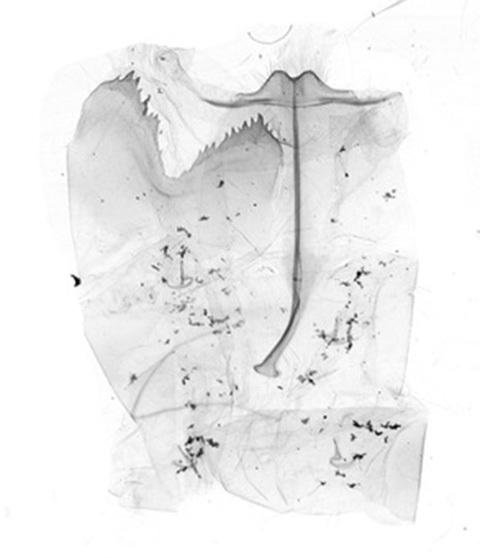
*Benbowia camilla*, ♂, paratype, Vietnam, Left: 8^th^ tergite, right: 8^th^ sternite, ASPC.
